# Orbital Cellulitis Complicated by Cerebral Vein Thrombosis and Meningitis in a Young, Healthy Adult

**DOI:** 10.7759/cureus.72770

**Published:** 2024-10-31

**Authors:** Sabrina Carpintieri, Elias Uyar, Alberto Gomez Veliz

**Affiliations:** 1 Medical School, Ross University School of Medicine, Miami, USA; 2 Internal Medicine, Ross University School of Medicine, Miami, USA; 3 Internal Medicine, Jackson Memorial Hospital, Miami, USA

**Keywords:** aseptic orbital cellulitis, atypical meningitis, cerebral venous sinus thrombosis (cvst), intracranial abscess, sphenoid sinusitis

## Abstract

Orbital cellulitis is a serious infection that can lead to severe complications if not promptly diagnosed and treated. This case report describes an unusual presentation of bilateral orbital cellulitis in a previously healthy 29-year-old female, complicated by cerebral vein thrombosis and meningitis. The patient's young age and lack of significant medical history make this case particularly noteworthy. Initially presenting with a local infection over the right nose that rapidly progressed to bilateral orbital cellulitis, subsequent imaging revealed extensive complications including bilateral cavernous sinus thrombosis, sphenoid sinusitis, and intracranial abscesses. Blood cultures were positive for methicillin-resistant Staphylococcus aureus (MRSA), an uncommon finding in community-acquired orbital cellulitis. The patient underwent sinus endoscopy and bilateral sphenoidotomy, followed by a prolonged course of intravenous and oral antibiotics. This case highlights the potential for severe complications in orbital cellulitis, even in young, healthy individuals, and emphasizes the importance of prompt, aggressive treatment and multidisciplinary management. It is imperative for clinicians to maintain a high index of suspicion for atypical pathogens and rare complications in seemingly routine cases.

## Introduction

Orbital cellulitis, or postseptal cellulitis, is a serious infection of the soft tissues surrounding the eye, posterior to the orbital septum [1]. While it can affect individuals of any age, it is particularly concerning when it occurs in young, otherwise healthy adults. This condition is more commonly observed in the pediatric population, making its occurrence in adults especially noteworthy [2]. This case report presents an unusual and severe instance of bilateral orbital cellulitis in a 29-year-old female with no significant medical history. The case is notable for its rapid progression from a localized nasal infection to extensive intracranial involvement, including cerebral vein thrombosis and meningitis.

The diagnosis of orbital cellulitis primarily relies on clinical findings, with key features including ophthalmoplegia, pain with eye movement, and proptosis. Imaging modalities such as CT and MRI can confirm the diagnosis and assess the extent of involvement. A recent analysis of microbial cultures from orbital abscesses and sinus samples revealed that Staphylococcus species were the predominant pathogens. Notably, 36% of the 22 positive Staphylococcal isolates demonstrated resistance to methicillin, while Streptococcus species were the second most frequently identified organisms [3]. The study also found that certain anaerobic bacteria, though less common, were sometimes implicated in infections, particularly in cases related to bite injuries from humans or animals, including Peptococcus, Peptostreptococcus, and Bacteroides. The identification of methicillin-resistant Staphylococcus aureus (MRSA) as the causative organism in this community-acquired infection of this patient adds another layer of complexity to the case. Around 10% of patients suffer vision impairment such as blindness, and this condition can lead to life-threatening complications such as meningitis, brain abscesses, and in rare cases, mortality [4].

Treatment of orbital cellulitis typically involves broad-spectrum antibiotics aimed at covering common pathogens, including MRSA, Streptococcus species, and gram-negative bacilli. In cases of suspected intracranial extension, coverage for anaerobic organisms is also crucial. Multidisciplinary management, often involving ophthalmologists and otolaryngologists, is essential for proper examination and to determine if surgical intervention is necessary.

This report aims to highlight the potential for severe complications in orbital cellulitis, even in seemingly low-risk individuals, and emphasize the importance of prompt, thorough diagnostic imaging and aggressive management.

## Case presentation

A 29-year-old female with no significant past medical history presented in August 2024 as a transfer from another hospital for an ophthalmology consultation and management of bilateral orbital cellulitis, with the right eye more affected than the left. The patient's young age and lack of medical history made this presentation particularly unusual, prompting a thorough investigation.

The patient's history revealed the onset of a local infection over the right nose approximately one week prior to presentation, which progressed to bilateral orbital cellulitis. This progression raised concerns about potential underlying factors or atypical pathogens.

The initial physical examination revealed visual acuity of 20/25 in both eyes. Anterior exam (Table 1) and fundus exam (Table 2) were performed at the bedside. Intraocular pressures were elevated at 23 mmHg in the right eye and 21 mmHg in the left eye. Pupils were anisocoric (right 4 mm, left 2 mm) but reactive to light with no afferent pupillary defect. Extraocular movements were significantly impaired, particularly in the right eye, with limitations in all directions of gaze. The patient denied any history of sinus infections, congestion, or rhinorrhea and did not use contacts or glasses. The absence of typical risk factors for orbital cellulitis, such as recent sinus infections or trauma, further added to the uniqueness of this case.

**Table 1:** Anterior segment exam upon initial presentation.

Structure/Abnormality to be observed	Right	Left
Visual Acuity	20/25	20/25
Lids/Lashes	Severe ptosis, mild crusting	Normal
Conjunctiva/Sclera	1-2+ chemosis inferiorly with injection inferiorly	White and quiet
Cornea	Clear	Clear
Anterior Chamber	Deep and quiet	Deep and quiet
Iris	Hyperemic	Clear
Lens	Clear	Clear
Vitreous	Clear	Clear
Pupil Size	4mm	2mm
Pupil Shape	Round	Round
Pupil Light Reflex	Poorly reactive due to orbital inflammation	Brisk and reactive
Anisocoria	4mm → 3.5mm (light to dark)	2mm → 3mm (light to dark)

**Table 2:** Fundus exam upon initial presentation.

Structure/Abnormality to be observed	Right	Left
Disc	Pink perfused, mild elevation and trace blurring of inferior and nasal borders of disc	Pink perfused, mild elevation and trace blurring of inferior and nasal borders of disc, trace blurring of superior border
Macula	Normal	Normal
Vessels	Normal	Normal
Periphery	Normal	Normal

The patient was initially treated empirically with intravenous vancomycin at the prior hospital. Upon transfer, the treatment plan was expanded to include metronidazole and cefepime, along with erythromycin and moxifloxacin eye drops. This broad-spectrum approach was chosen due to the severity of the presentation and the unknown etiology at that time.

The following day, the patient developed significant leukocytosis with a white blood cell count of 20,000. Surprisingly, blood cultures were positive for MRSA, an uncommon finding in community-acquired orbital cellulitis. This finding prompted a reevaluation of the treatment approach and raised questions about potential underlying immunological factors.

**Figure 1:**
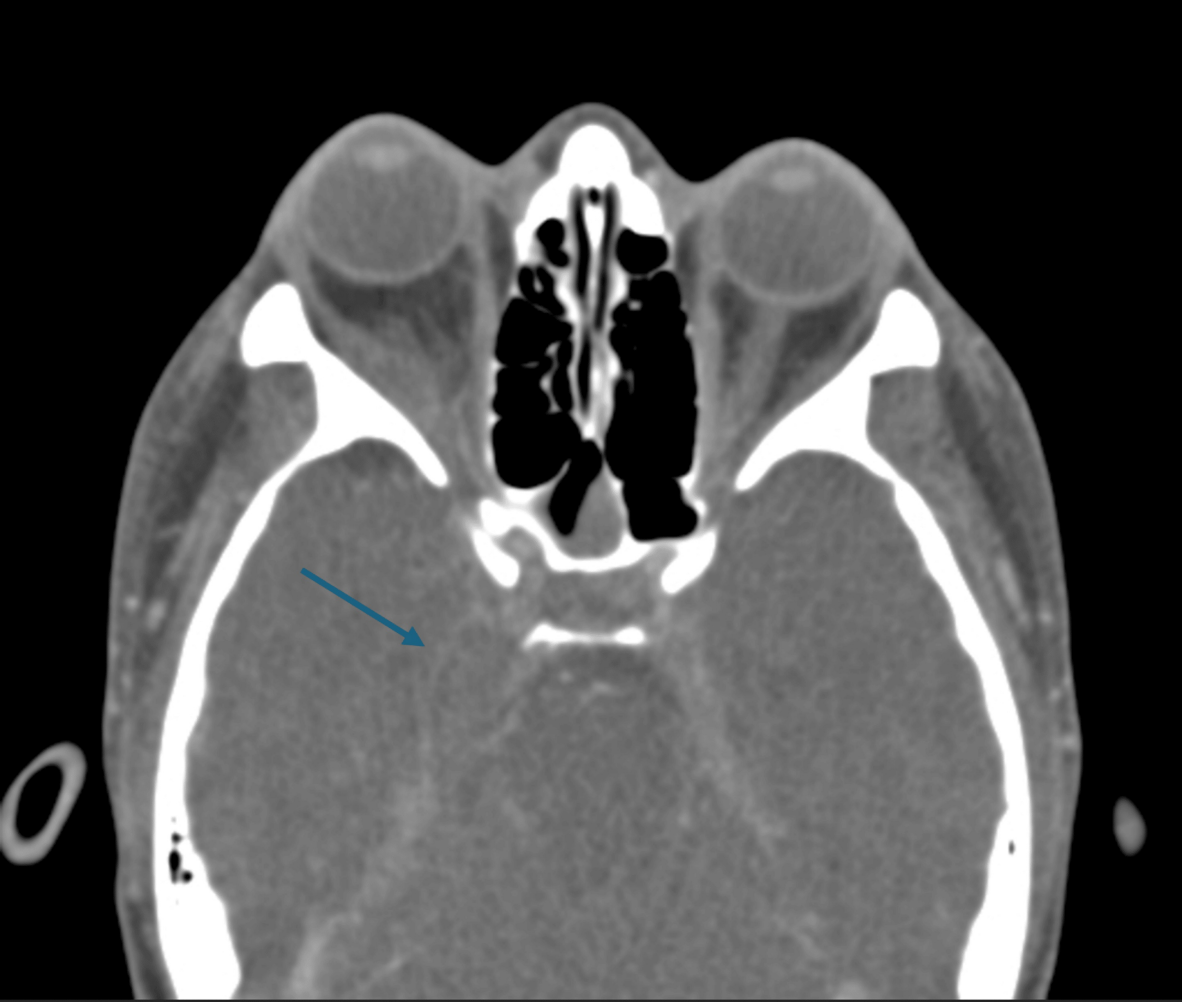
Axial CT scan of the superior orbit. The blue arrow points to the peripheral enhancing collection along the medial aspect of the right middle cranial fossa, measuring at least 3.2 cm x 1.4 cm.

**Figure 2:**
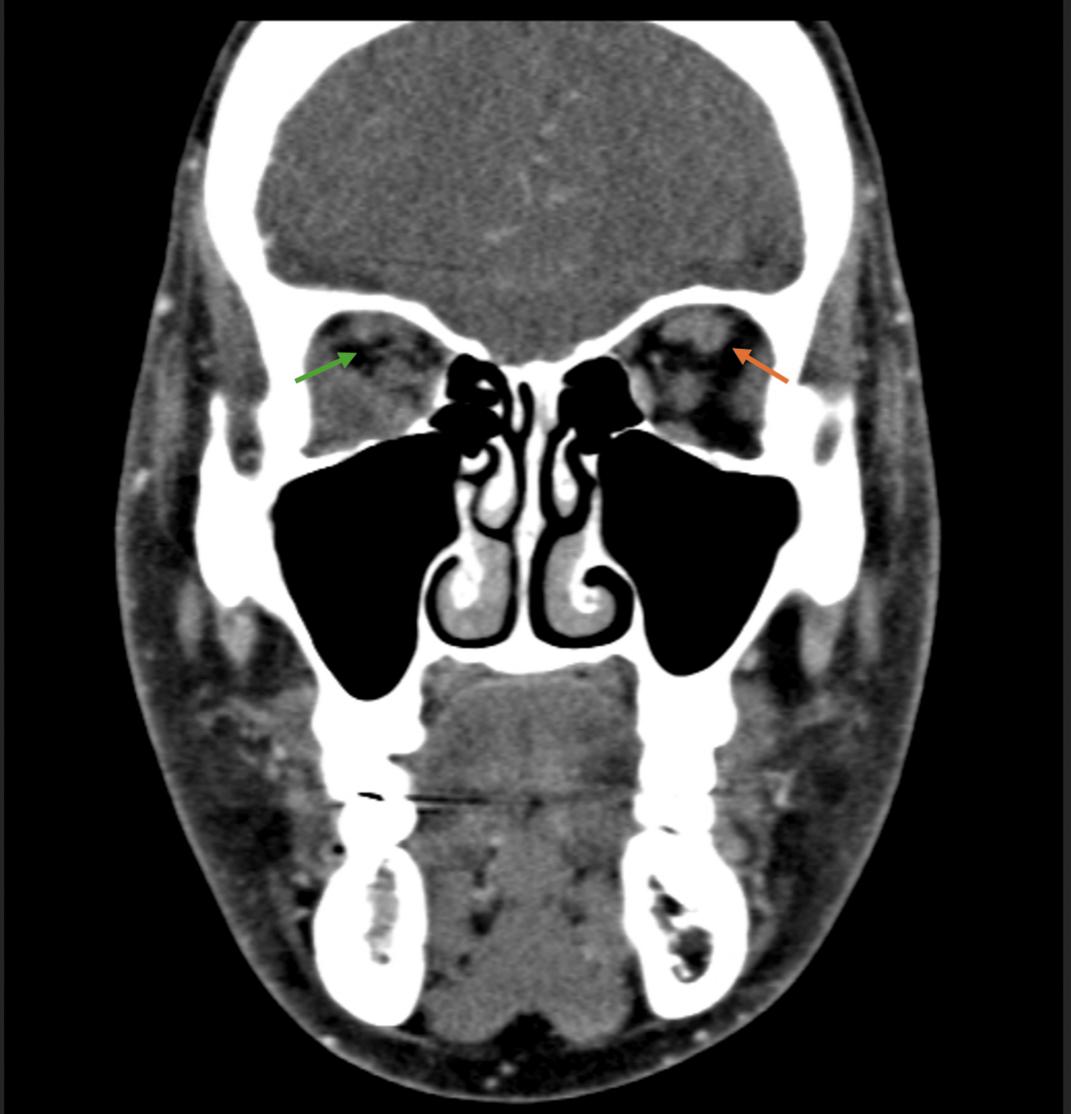
Coronal CT scan of the superior orbit. The green arrow shows decreased enhancement involving the right superior ophthalmic vein, concerning for thrombosis. The orange arrow points to a focal dilation and filling defect involving the left infraorbital and left superior ophthalmic vein, consistent with superior ophthalmic vein thrombosis.

Prior to imaging, the patient was initiated on prophylactic low molecular weight heparin therapy to mitigate the risk of cerebral venous sinus thrombosis. CT of the face with contrast revealed extensive complications, including thrombosis of the bilateral superior ophthalmic veins, asymmetric thickening and enhancement of the right cavernous sinus, and a peripheral enhancing collection in the middle cranial fossa, as shown below (Figures 1-2). These findings were unexpected, highlighting the potential for severe complications even in seemingly low-risk individuals.

**Figure 3:**
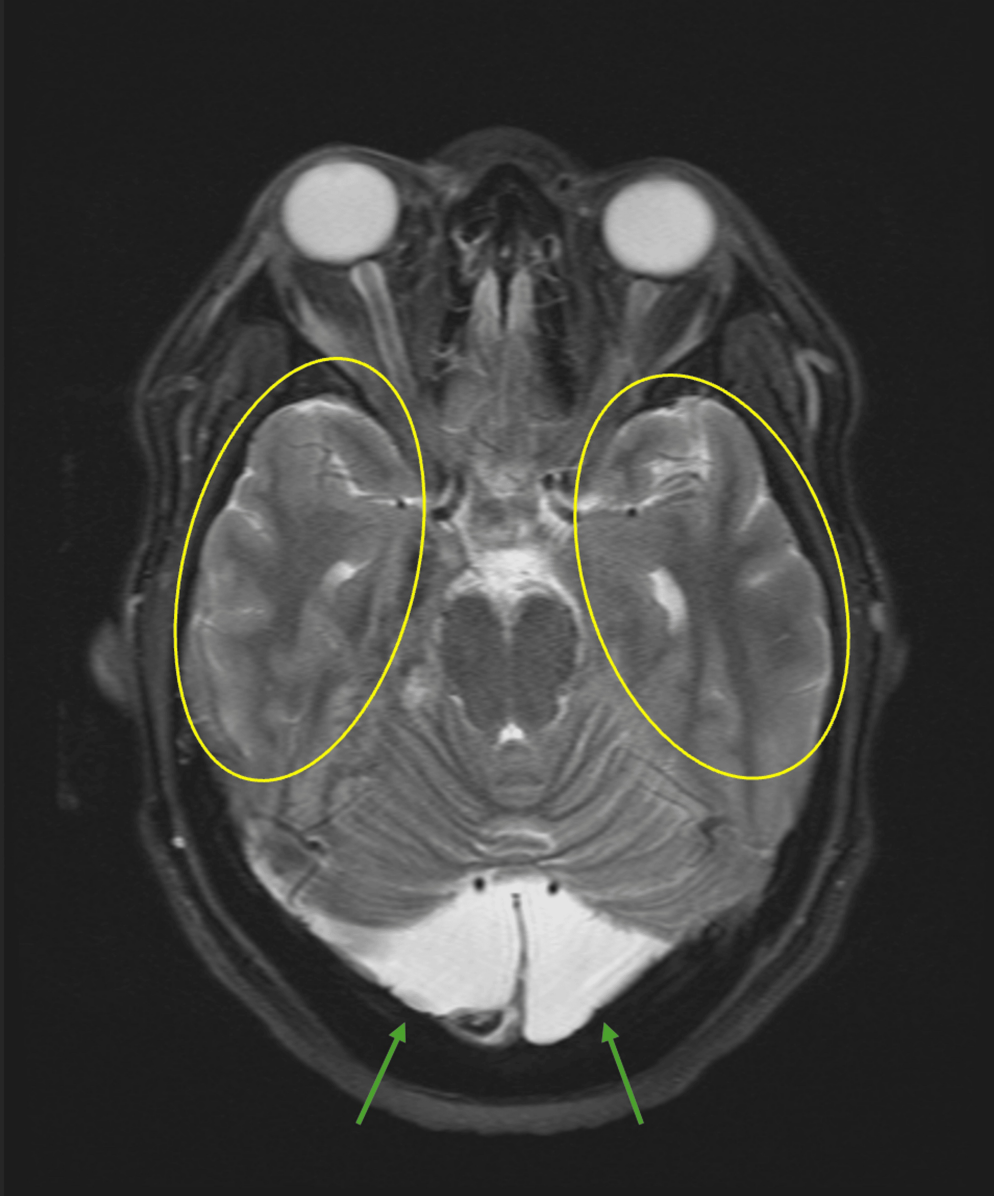
MRI brain: T2-weighted. The yellow circles highlight extensive leptomeningeal enhancements involving the anterior aspects of the posterior fossa. The green arrows point to a CSF intensity area in the posterior cranial fossa measuring 3.7 x 5.9 x 4.9 cm, which is favored to represent a mega-cisterna magna.

**Figure 4:**
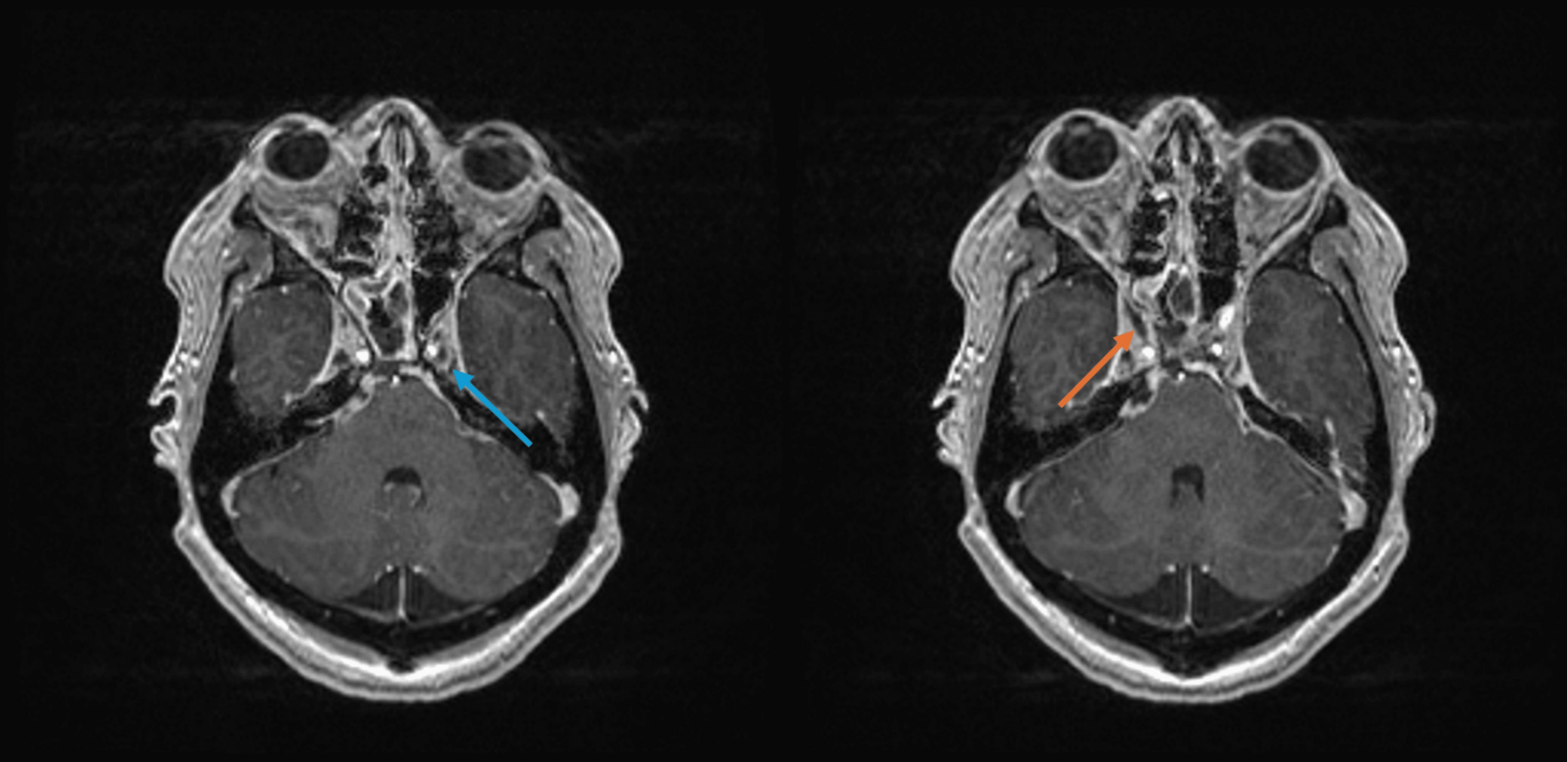
MRI brain: T1-weighted. The blue arrow points to an area with a lack of enhancement within the left cavernous sinus, while the orange arrow points to small filling defects within the right cavernous sinus, measuring approximately 1.3 cm x 0.4 cm. There is diffuse heterogeneous enhancement throughout the cavernous sinuses with asymmetric thickening on the right compared to the left.

MRI of the brain was performed to further evaluate and better delineate the findings suggested by the initial CT, providing superior soft tissue contrast and more detailed visualization of the intracranial structures. MRI of the brain showed extensive leptomeningeal enhancement, and the lack of CSF suppression of FLAIR within the posterior cerebral sulci raised concern for meningitis, seen in Figure 3. MRI of the orbits revealed evidence of bilateral papilledema with enhancement of the optic nerve heads suggestive of papillitis, as well as thrombosis of the bilateral superior ophthalmic veins and partial thrombosis of the bilateral cavernous sinuses seen in Figure 4. The extent of these complications demonstrates the importance of thorough imaging in cases of orbital cellulitis, even when the initial presentation might not suggest such extensive involvement.

Due to the severity of imaging findings, the patient underwent sinus endoscopy and bilateral sphenoidotomy. Intraoperative findings included polypoid mucosa and thick, fibrinous, purulent material in the right sphenoid sinus, while the left sphenoid sinus was without mucosal disease.

Post-operatively, the patient showed improvement in right eye proptosis, periorbital swelling, and extraocular movements, though not completely resolved. Follow-up physical examination showed improved visual acuity and intraocular pressures, with persistent anisocoria and extraocular movement limitations. The gradual improvement highlighted the importance of aggressive surgical intervention in conjunction with medical management.

The patient’s blood cultures became negative 24 hours later, and her condition improved. She became afebrile, and plans were made for discharge with weekly ophthalmology follow-ups and infectious disease consultations. The treatment plan included continuing intravenous vancomycin for MRSA for two more weeks from the negative cultures, followed by oral linezolid for another two weeks. Extended oral therapy with doxycycline was considered if there were concerns for persistent staphylococcal infection. This prolonged and carefully tailored antibiotic regimen reflects the complexity of managing severe orbital cellulitis with atypical pathogens. The patient was to continue the same dosage of heparin given in the hospital. It will be decided at the follow-up with ophthalmology if the patient should be switched to direct oral anticoagulants such as rivaroxaban or apixaban, which would not require regular INR monitoring. The patient will be on anticoagulation for approximately the next 3 months, but the duration can change depending on the progress of the patient. A follow-up MRI or CT venography will be scheduled in the next 6-12 weeks to assess the resolution of the thrombosis.

This case illuminates several critical aspects of orbital cellulitis management. The potential for severe complications, even in individuals without typical risk factors, challenges clinicians to maintain vigilance regardless of patient demographics. The presence of MRSA in this community-acquired infection emphasizes the importance of considering atypical pathogens in all cases of orbital cellulitis. Furthermore, this case demonstrates the crucial role of early and extensive imaging in identifying complications that may not be clinically apparent. The successful management of this complex case highlights the value of a multidisciplinary approach, involving ophthalmology, neurology, infectious disease, and otolaryngology specialists. It also reinforces the need for aggressive surgical intervention when indicated and the importance of tailoring long-term antibiotic therapy based on culture results and clinical response.

In conclusion, this case serves as a reminder to clinicians to maintain a high index of suspicion for severe complications and atypical pathogens in orbital cellulitis, regardless of the patient's age or health status. The importance of prompt diagnosis, aggressive treatment, and multidisciplinary management is essential in ensuring optimal outcomes in such complex cases.

## Discussion

This case of bilateral orbital cellulitis in a young, healthy adult presents several noteworthy aspects that warrant discussion. The atypical presentation and rapid progression from a localized nasal infection to bilateral orbital cellulitis with intracranial complications in a seemingly low-risk patient demonstrate the potential virulence of the causative organism and the need for high clinical suspicion. The identification of MRSA in blood cultures, uncommon in community-acquired orbital cellulitis, highlights the changing epidemiology of antibiotic-resistant organisms and the need to consider atypical pathogens in all cases.

CT and MRI played a crucial role in identifying the extent of the infection and associated complications, with MRI providing superior soft tissue contrast and more detailed visualization of intracranial structures. Thorough imaging in cases of orbital cellulitis may often be overlooked but tends to make the management of these cases much simpler, even when the initial presentation might not suggest extensive involvement.

Successful management of this complex case involved collaboration between multiple specialties, including ophthalmology, neurology, infectious disease, and otolaryngology. The decision to perform sinus endoscopy and bilateral sinus sphenoidotomy highlights the importance of aggressive surgical intervention when indicated, even in young patients. Surgical drainage or decompression of the orbital cavity is a common technique if orbital pressure remains elevated. An adult patient with a similar presentation of unilateral orbital pain and reduced visual acuity underwent sphenoidotomy after failing to respond to intravenous antibiotics [5]. Following the surgical procedure, her vision returned to normal. The post-operative improvement in our patient's condition validates this approach.

Given the prevalence of methicillin-resistant infections, empiric therapy often includes vancomycin as a preferred first-line agent. This is typically complemented with medications such as cefotaxime for Gram-negative coverage, and either metronidazole or clindamycin to address potential anaerobic pathogens [6]. While some regions may favor cephalosporins and aminoglycosides [7], the increasing trend of resistance to fluoroquinolones reported in recent studies emphasizes the need for judicious antibiotic selection based on local epidemiology [8].

The role of corticosteroids in orbital cellulitis treatment remains controversial. Current evidence is insufficient to definitively support their use, highlighting the need for more clinical trials [9]. A recent meta-analysis and literature review found that the use of systemic steroids as an adjunct to systemic antibiotics for orbital cellulitis can reduce orbital inflammation with minimal risk of worsening infection [10]. A careful approach balances potential benefits against risks in managing this serious condition. The prolonged and carefully tailored antibiotic regimen, including intravenous vancomycin followed by oral linezolid, reflects the complexity of managing severe orbital cellulitis with atypical pathogens. Linezolid was added to the therapy because it is highly effective against MRSA and achieves good tissue penetration, including the orbital and CNS regions, which is important considering the patient's complication of a cavernous sinus thrombus. Naesens R et al. reviewed 12 cases of community-acquired MRSA (CA-MRSA) with CNS involvement, including conditions like brain abscesses and cavernous sinus thrombosis. The review noted that patients treated with linezolid showed better outcomes than those treated with vancomycin, suggesting that linezolid may be more effective in managing severe CNS infections caused by CA-MRSA [11]. The consideration of extended oral therapy with doxycycline demonstrates the need for ongoing vigilance and readiness to adjust treatment based on clinical response.

The plan for weekly ophthalmology follow-ups and infectious disease consultations emphasizes the importance of close monitoring in such complex cases, even after initial improvement and discharge. However, it is important to acknowledge the limitations of this single case report. Treatment outcomes may vary significantly across different patient populations based on factors such as age, comorbidities, immune status, and local resistance patterns. Additionally, the aggressive surgical and medical approach described here may not be universally applicable or necessary for all cases of orbital cellulitis. While the successful outcome in this case is encouraging, it should be interpreted within the context of these limitations. Further research, particularly prospective studies with larger patient cohorts, would be valuable in establishing more definitive treatment guidelines and identifying predictors of disease progression and treatment response. This case serves as a valuable reminder of the potential severity of orbital cellulitis and the importance of a comprehensive, aggressive approach to diagnosis and management, regardless of the patient's age or prior health status.

## Conclusions

This case report of bilateral orbital cellulitis complicated by cerebral vein thrombosis and meningitis in a young, healthy adult highlights several crucial lessons for clinicians. It demonstrates that severe complications of orbital cellulitis can occur even in patients without typical risk factors, emphasizing the need for vigilance in all cases. The successful management of this complex case through a multidisciplinary approach, including aggressive surgical intervention and tailored antibiotic therapy, provides a model for handling similar cases in the future. This report serves as a reminder to maintain a high index of suspicion for severe complications and atypical pathogens in cases of orbital cellulitis, regardless of the patient’s age or health status. Prompt diagnosis, thorough imaging, aggressive treatment, and multidisciplinary management ensure optimal outcomes in complex cases of orbital cellulitis.
